# Supplementary figures and images for: Distinct tooth regeneration systems deploy a conserved battery of genes
Source: EvoDevo. 2021 Mar 25;12:4. doi: 10.1186/s13227-021-00172-3 (PMC7995769; doi:10.1186/s13227-021-00172-3)

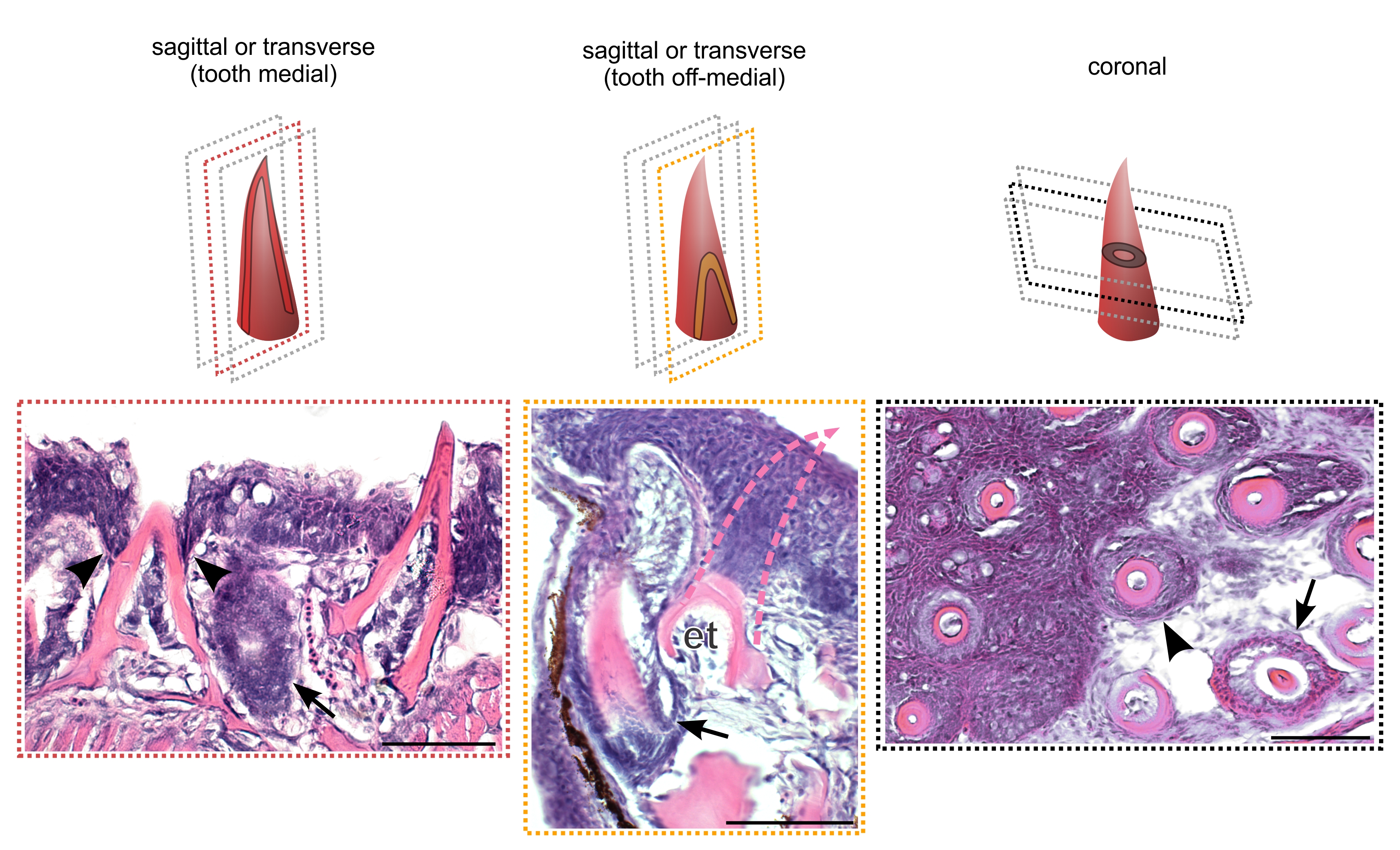

Supplement: Supplementary file 1 — Additional file 1: Fig. S1. Histological plane orientation and description in sticklebacks. Illustrations at top depict a tooth (red cone) and indicate section planes with dotted rectangles, which are pictured below for each respective plane of section (labeled at top). These planes of section roughly translate to whole-animal anatomical planes. Left and middle illustrations show the sagittal and/or transverse plane (because stickleback teeth are mostly conical, these two planes of section appear generally similar in section). On these planes of section, teeth often appear off their medial axis (left vs center illustration, see labels). Rightmost illustration depicts the coronal plane, where teeth appear as rings of bone. Bottom images are of H&E-stained sections representing each illustrated plane of section above. Left bottom image shows a pharyngeal tooth on the transverse plane. Center bottom image shows an oral tooth on the sagittal plane. Right bottom image shows a pharyngeal tooth sectioned on the coronal plane. An erupted tooth (“et”) is indicated in the center image of an off-medial tooth (suggested with a red dotted line) sectioned on the sagittal plane. In all H&E images, tooth germs are indicated with an arrow, and the SDE is indicated with arrowheads. All scale bars = 50 μm. [file 13227_2021_172_MOESM1_ESM.jpg]

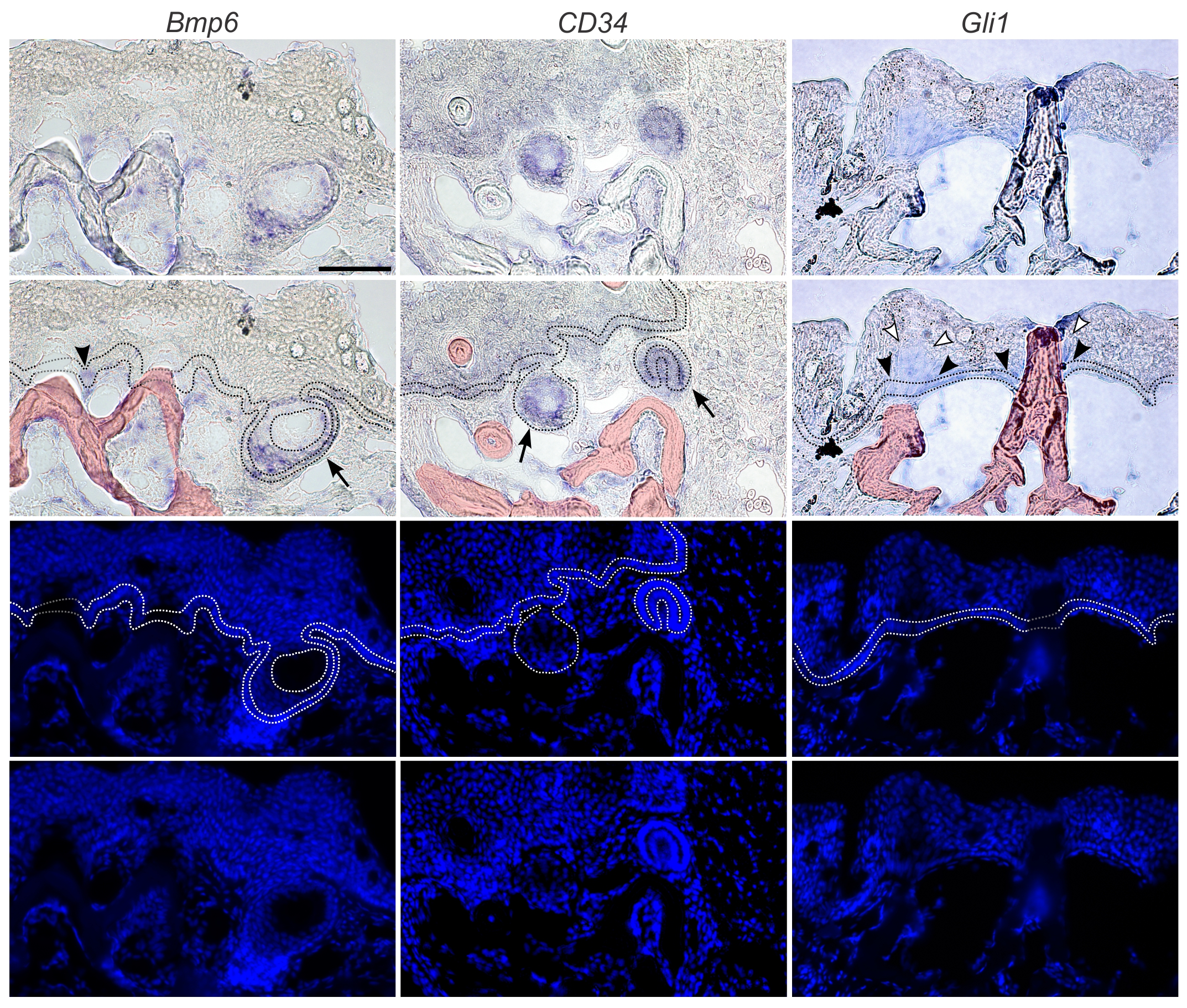

Supplement: Supplementary file 2 — Additional file 2: Fig. S2. DAPI counterstaining in situ hybridized sections aids in distinguishing epithelium from mesenchyme. Three gene expression patterns are shown as examples, labeled at top of columns in figure. Each column shows the same section corresponding to each gene, top two images are in brightfield, bottom two show DAPI fluorescence. 1st row of brightfield images are shown without any markup. 2nd row of brightfield images outline the basalmost epithelium with black dotted lines where possible (gray segments suggest borders out of the plane of section); bone is false-colored red. Expression domains in tooth germs are indicated with black arrows. Expression in the SDE is indicated with black arrowheads. Superficial epithelium with Gli1 expression adjacent to erupted teeth is indicated with white arrowheads. 3rd row of images shows the DAPI counterstain of each brightfield image above it, with the same epithelium outlines overlain (white dotted lines). 4th row shows DAPI images without any markup. Scale bar = 50 μm and applies to all panels. [file 13227_2021_172_MOESM2_ESM.jpg]

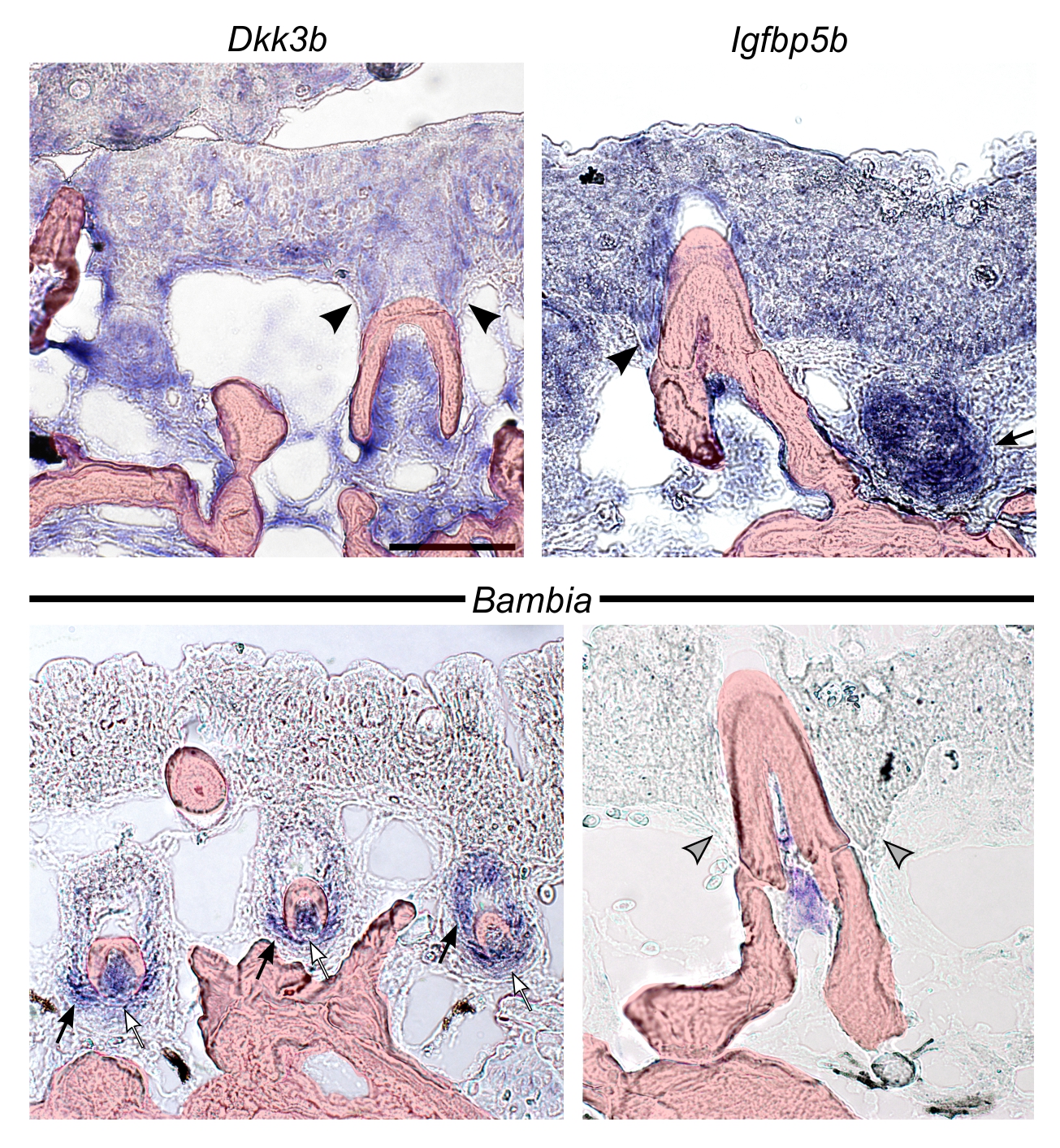

Supplement: Supplementary file 3 — Additional file 3: Fig. S3. Stickleback Dkk3b, Igfbp5b, and Bambia ISH on sections through dental tissue. The stickleback SDE was positive for Dkk3b and Igfbp5b (black arrowheads in top images), though these genes marked many cell populations across the dental field and surrounding tissues. Bambia was detected in tooth germ epithelium (black arrows) and mesenchyme (white arrows), and additionally in mature tooth mesenchyme, but never in the SDE (gray arrowheads). Scale bar = 50 μm and applies to all panels. [file 13227_2021_172_MOESM3_ESM.jpg]

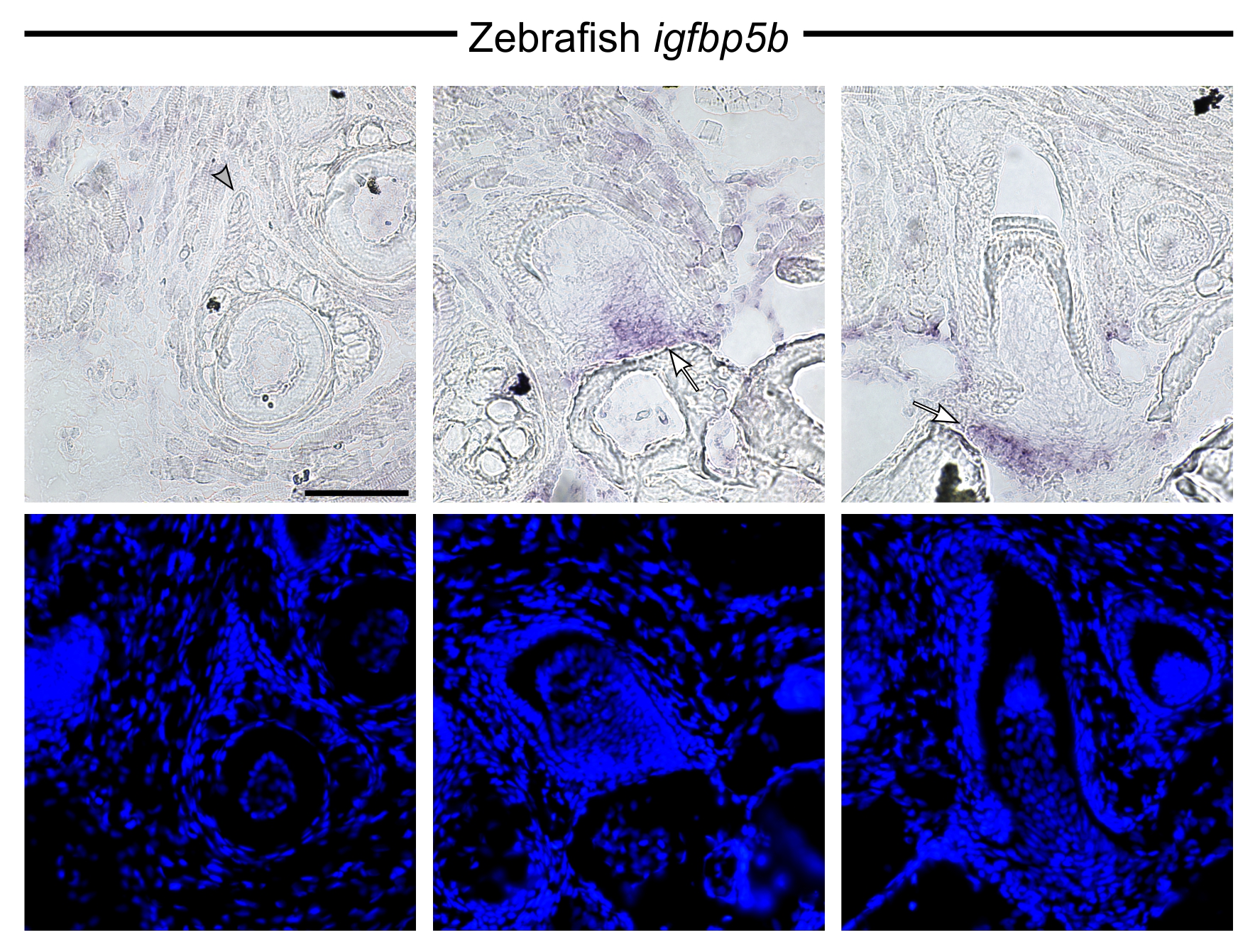

Supplement: Supplementary file 4 — Additional file 4: Fig. S4. Zebrafish igfbp5b expression in deep tooth mesenchyme. Top row shows brightfield images, bottom row shows the corresponding DAPI counterstains to each brightfield image above. The SDL of zebrafish did not show igfbp5b expression (gray arrowhead). Expression was detected in a subset of the deep dental mesenchyme (white arrows). Scale bar = 50 μm and applies to all panels. [file 13227_2021_172_MOESM4_ESM.jpg]

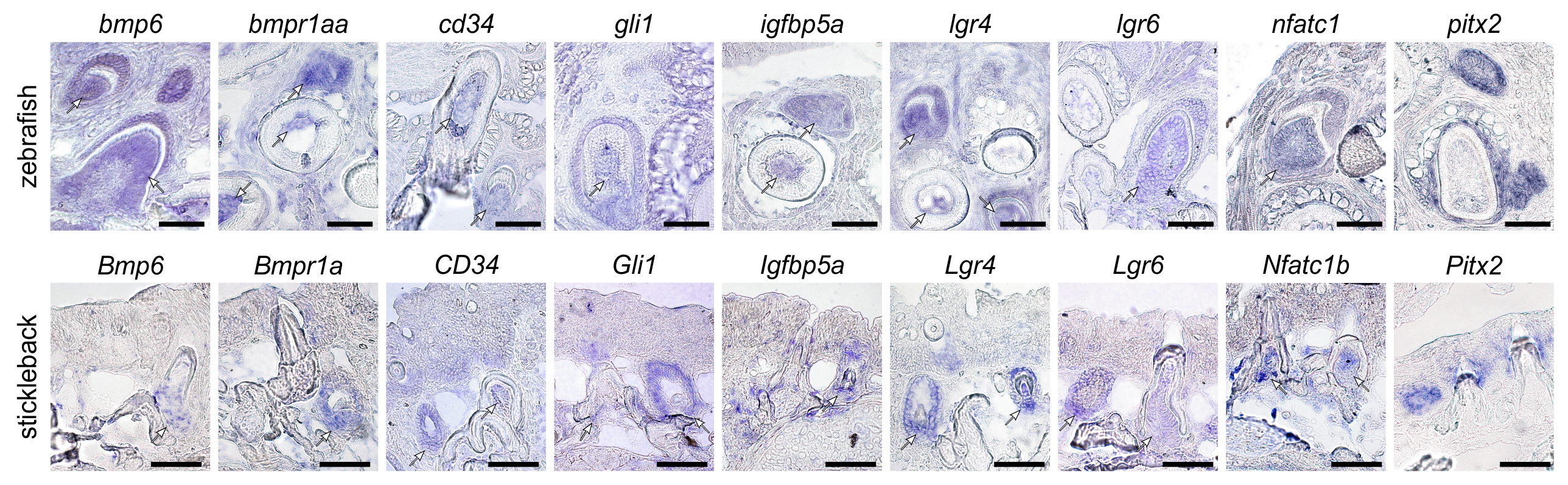

Supplement: Supplementary file 5 — Additional file 5: Fig. S5. Expression of Bmp6, Bmpr1a, CD34, Gli1, Igfbp5a, Lgr4, Lgr6, Nfatc1, and Pitx2, highlighting mesenchymal expression. All of these genes were found to mark the SDE, except for Pitx2, and are expressed in some portion of dental mesenchyme (white arrows). Species and gene assayed are indicated in the figure. All scale bars = 50 μm. [file 13227_2021_172_MOESM5_ESM.jpg]
